# Malignant sigmoid-duodenal fistula: case report and review of the literature

**DOI:** 10.1186/1471-2318-11-S1-A36

**Published:** 2011-08-24

**Authors:** V Minutolo, A Buttafuoco, G Gagliano, O Minutolo, R Lanteri, A Racalbuto, A Licata

**Affiliations:** 1 University of Catania (Italy) Department of Surgical Sciences, Organ Transplantation and Advanced Technologies. Via Santa Sofia 84, Catania, Italy

## Background

Colonic-duodenal fistulas are rare, and may be secondary to benign or malignant conditions. Malignant duodenocolonic fistulas may also develop in patients with right colon or hepatic flexure carcinoma or duodenal malignancy. The sigmoido-duodenal malignant fistula is exceptional and, to our knowledge, only two previous cases have been reported. The first case was treated in our Institution in 1981 and published by Russello [1] and the second was reported by Melissas in 2002 [2] in the literature. We present the third case of malignant fistula between the duodenum and sigmoid colon in a 84-years-old male patient.

## Case report

**Figure 1:**
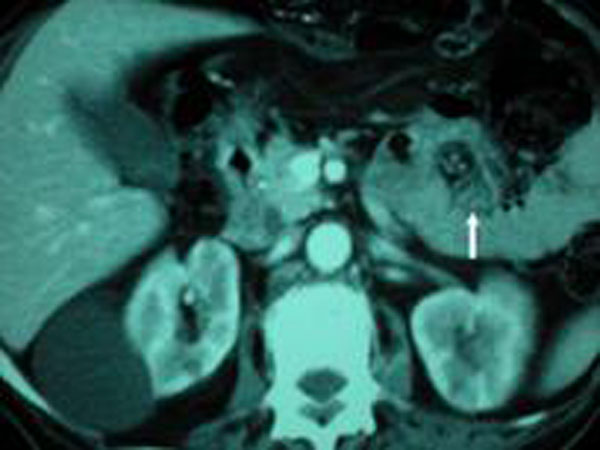
CT scan

**Figure 2:**
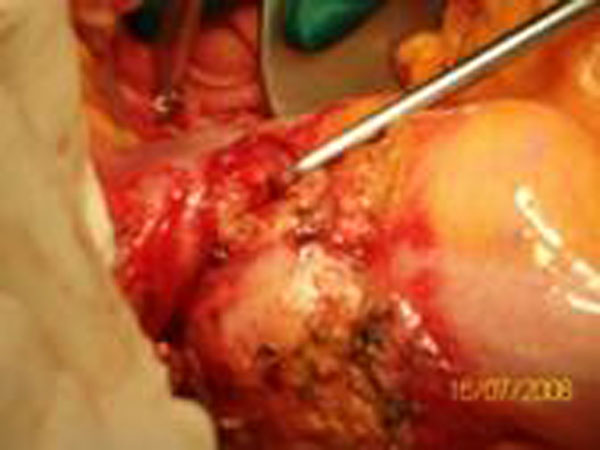
Duodenal neoplastic involvement

An 84-year-old white male was observed in our department. He came to his general practitioner with anaemia. He had an eight month history of diarrhoea and constipation, poor appetite, weight loss, nausea and vomiting. Blood tests revealed: a) two tumour markers outside normal range: CEA: 18 ng/ml e CA 19-9: 121U/ml; b) severe anaemia: Hb=7,0 gr/dl, RBC=3,23 mil/uL, Ferritin=4 ng/ml. The colonscopy revealed an ulcerated tumor about 25 cm from the anal verge obstructing the sigmoid lumen and not allowing the passage of the instrument. The fistula was not identified at by endoscopy. The CT scan of the abdomen did not show the fistula and did not highlight any local spread or distant metastasis. The neoplastic lesion of the sigma of about 8 cm in length and 6 cm in thickness with tight luminal eccentric stenosis and dilatation of the colon upstream was highlighted (Figure 1). At laparotomy, the sigmoid colon, because of a dolicocolon, was found to be adhered to the fourth part of the duodenum where the fistula was located. The patient had a surgical resection of the affected portion of the sigmoid colon and resection of the fourth duodenal portion (Figure 2). En bloc resection of sigmoid colon and duodenum respecting the anatomical margins and obtaining radical resection of the cancer was achieved. An end-to-end colorectal anastomosis was done using a circular stapler and an end-to-side duodenojejunal anastomosis using a manual suture. He did not have any post-operative complications. Hystology demonstrated a primary adenocarcinoma of the colon, mucinous type, infiltrating the duodenum: pT4 N0 M0, Stage II, Dukes’ B. The resection margins were free of disease. The patient has not received adjuvant chemotherapy and after twelve months is alive and in good health, free from recurrent disease.
